# The role of omega-3 polyunsaturated fatty acids in the non-surgical management of periodontitis: a systematic review and meta-analysis

**DOI:** 10.3389/froh.2026.1761032

**Published:** 2026-03-13

**Authors:** Gianluca Benincasa, Margherita Giorgia Liguori, Francesco Tarallo, Sabina Saccomanno, Leonardo Mancini, Enrico Marchetti

**Affiliations:** 1 Department of Life, Health and Environmental Sciences, University of L’Aquila, L’Aquila, Italy; 2 Clinic of Reconstructive Dentistry, Center of Dental Medicine, University of Zurich, Zurich, Switzerland; 3 Department of Life Science, Health and Health Professions, Link Campus University, Rome, Italy

**Keywords:** acetylsalicylic acid, host modulation therapy, meta-analysis, non-surgical periodontal therapy, omega-3 fatty acids, periodontitis, EPA, DHA

## Abstract

**Aim:**

Omega-3 polyunsaturated fatty acids (PUFA) possess anti-inflammatory and pro-resolving properties, suggesting potential benefits as adjuncts to non-surgical periodontal therapy (NSPT). This systematic review and meta-analysis aimed to evaluate the clinical efficacy of Omega-3 PUFA supplementation, with or without acetylsalicylic acid (ASA), in patients with periodontitis.

**Methods:**

Electronic and manual searches were conducted across MEDLINE (PubMed), the Cochrane Central Register of Controlled Trials, and Google Scholar up to September 2025. Randomized controlled trials (RCTs) assessing NSPT combined with Omega-3 supplementation for ≥1 month were included. Primary outcomes were changes in clinical attachment level (CAL) and probing pocket depth (PPD); data were pooled using random-effects models, and risk of bias was assessed with the Cochrane RoB 2 tool.

**Results:**

Sixteen RCTs met the inclusion criteria, and nine were included in the quantitative synthesis. Meta-analysis revealed significant improvements in CAL [mean difference (MD) = −0.49 mm; 95% CI −0.75 to −0.23] and PPD (MD = −0.44 mm; 95% CI −0.62 to −0.25) at 3 months, favoring NSPT + Omega-3 over NSPT alone. At 6 months, differences remained favorable and statistically significant for both CAL (MD = −0.58 mm; 95% CI −0.96 to −0.21) and PPD (MD = −0.45 mm; 95% CI −0.76 to −0.14).

**Conclusions:**

Adjunctive Omega-3 PUFA supplementation improves short-term periodontal healing following NSPT, yielding modest but clinically meaningful benefits in CAL and PPD. Despite promising results, further standardized RCTs are needed to confirm long-term efficacy and clarify the potential synergistic role of ASA co-administration.

**Systematic Review Registration:**

https://www.crd.york.ac.uk/PROSPERO/view/CRD42024508208, PROSPERO CRD42024508208.

## 1 Introduction

Periodontitis is the sixth most prevalent disease worldwide and remains the leading cause of tooth loss in adults (1). It is a chronic, multifactorial inflammatory condition initiated by bacterial biofilms and modulated by host immune and genetic factors in combination with environmental and lifestyle influences, ultimately leading to the progressive destruction of periodontal supporting tissues (2). Inflammation represents a physiological host defense mechanism against infection and injury (3); however, when dysregulated, it contributes to chronic tissue damage and impaired resolution of the inflammatory process.

Host modulation therapy has been proposed as an adjunctive approach to conventional periodontal treatment, aiming to attenuate the destructive aspects of the host response while promoting the resolution of inflammation and tissue repair (4). Among the pharmacological agents investigated, Omega-3 polyunsaturated fatty acids (PUFA) and acetylsalicylic acid (ASA) have received considerable attention due to their regulatory effects on lipid mediator pathways. Omega-3 fatty acids are incorporated into cell membrane phospholipids and serve as precursors of bioactive lipid mediators that influence intracellular signaling, gene transcription, and the course of inflammatory processes (5).

Docosahexaenoic acid (DHA) and eicosapentaenoic acid (EPA), the principal Omega-3 fatty acids, are substrates for the biosynthesis of specialized pro-resolving mediators (SPM)—including resolvins, protectins, and maresins—that actively terminate inflammation and promote tissue regeneration (6–10). Conversely, arachidonic acid–derived eicosanoids such as leukotrienes (LT) and prostaglandins (PG) are well recognized for their pro-inflammatory roles (11–14). Lipoxins (LX) and their analogs, aspirin-triggered lipoxins [15-epi-LX, also called aspirin-triggered lipoxins (ATL)], are potent counter-regulators of PMN-mediated injuries and acute inflammation (15–17). Aspirin modulates these pathways by acetylating cyclooxygenase-2 (COX-2), thereby inhibiting prostaglandin synthesis and inducing ATL generation, which enhances endogenous mechanisms of inflammation resolution (6, 9, 17–23).

On this biochemical basis, the adjunctive administration of Omega-3 PUFA and ASA in combination with subgingival instrumentation could theoretically reduce periodontal tissue destruction by promoting pro-resolving lipid mediator activity. Although the European Federation of Periodontology (EFP) S3 clinical guidelines currently do not recommend Omega-3 PUFA supplementation for the management of stage I–III periodontitis (24) due to insufficient supporting evidence, recent systematic reviews and meta-analyses (25–29) have suggested that Omega-3 PUFA may improve clinical outcomes when used as an adjunct. These emerging data support a potential paradigm shift in periodontal therapy, emphasizing the active resolution of inflammation rather than its mere suppression (30).

Therefore, the present systematic review and meta-analysis aims to evaluate the effectiveness of Omega-3 PUFA therapy as an adjunct to subgingival instrumentation in the treatment of periodontitis. The null hypothesis states that supplementation with Omega-3 PUFA does not produce additional clinical benefits compared with subgingival instrumentation alone.

## 2 Methods

This review has been registered at the National Institute for Health Research PROSPERO, International Prospective Register of Systematic Reviews and has been assigned the number CRD42024508208. The systematic review with meta-analysis was conducted in accordance with the Cochrane Handbook for Systematic Reviews of Interventions (31).

### 2.1 PICO criteria definitions

#### 2.2.1 Patients

Adults (≥18 years), systemically healthy or with controlled systemic conditions, diagnosed with stage II–IV periodontitis according to the current classification.

#### 2.2.2 Intervention

Non-surgical periodontal therapy (NSPT) involving subgingival instrumentation supplemented with Omega-3 PUFA administration for a minimum duration of one month, with or without concomitant use of ASA.

#### 2.2.3 Control

Non-surgical periodontal therapy with subgingival instrumentation supplemented with a placebo.

#### 2.2.4 Outcomes

##### 2.2.4.1 Primary outcomes

Clinical parameters of periodontal status, including changes in clinical attachment level (CAL) and probing pocket depth (PPD), assessed in both test and control groups.

##### 2.2.4.2 Secondary outcomes

Evaluation of the additional effect of combining omega-3 supplementation with ASA, and assessment of patient-reported outcome measures (PROMs) related to tolerability and adverse effects.

#### 2.2.5 Study design

Randomized controlled clinical trials (RCTs).

### 2.2 Focused question

In patients with periodontitis from stage II to stage IV, does supplementation with omega-3 polyunsaturated fatty acids, with or without acetylsalicylic acid, in addition to non-surgical periodontal therapy, improve clinical outcomes compared with non-surgical therapy alone?

### 2.3 Search strategy

The reporting of this systematic review and meta-analysis adhered to the PRISMA (Preferred Reporting Items for Systematic Reviews and Meta-Analyses) 2020 guidelines (32).

An electronic search was conducted to identify studies relevant to the objectives of this review across three databases: MEDLINE (PubMed), Cochrane Central Register of Controlled Trials, and Google Scholar. The search was performed between September 2023 and September 2025.

Keywords and MeSH terms related to “periodontitis”, “non-surgical periodontal therapy,” and “omega-3 polyunsaturated fatty acids” were combined using Boolean operators (AND, OR, NOT). Filters were applied to include studies conducted on human adults (≥18 years) and published in English. The relevant keywords were combined as follows for the search: ((“periodontal disease"[MeSH Terms] OR “periodontitis"[MeSH Terms] OR periodontitis[All Fields] OR “chronic periodontitis"[All Fields] OR “periodontal therapy"[All Fields] OR “periodontal treatment"[All Fields] OR “non-surgical periodontal therapy"[All Fields] OR “scaling and root planing"[All Fields] OR “subgingival instrumentation"[All Fields]) AND (“omega-3"[All Fields] OR “omega 3"[All Fields] OR “omega-3 fatty acids"[MeSH Terms] OR “polyunsaturated fatty acids"[All Fields] OR PUFA[All Fields] OR EPA[All Fields] OR DHA[All Fields] OR “eicosapentaenoic acid"[All Fields] OR “docosahexaenoic acid"[All Fields] OR “fish oil"[All Fields] OR “host modulation therapy"[All Fields] OR aspirin[All Fields] OR “acetylsalicylic acid"[All Fields]) AND (“randomized controlled trial"[Publication Type] OR randomized[All Fields] OR randomised[All Fields] OR “clinical trial"[All Fields])) NOT [“animals"[MeSH Terms] NOT “humans"[MeSH Terms]].

A manual search was additionally performed to identify relevant studies not captured by the electronic search. The reference lists of the included articles and related systematic reviews were screened to identify additional eligible trials published between 2010 and 2025. The manual search also included the following journals: *Journal of Periodontology*, *Journal of Clinical Periodontology*, *Clinical Oral Investigations*, and *Journal of Periodontal Research.* Two independent reviewers (GB and FT) screened all titles, abstracts, and full-text articles according to the predefined inclusion and exclusion criteria. Disagreements were resolved by discussion with a third reviewer (MGL) until consensus was achieved.

### 2.4 Inclusion criteria

Studies were included if they met all of the following criteria:

- Randomized controlled clinical trials (RCTs).
- Conducted on human subjects aged ≥18 years.
- Participants diagnosed with periodontitis, with stage and grade defined according to the current classification system whenever reported (24, 33–35).
- Intervention consisting of non-surgical periodontal therapy (NSPT) with subgingival instrumentation, combined with Omega-3 polyunsaturated fatty acid (PUFA) supplementation.
- Duration of Omega-3 supplementation ≥1 month.
- Reported clinical outcomes for both test and control groups, including at least clinical attachment level (CAL) and probing pocket depth (PPD).

### 2.5 Exclusion criteria

Studies were excluded if they met any of the following criteria:

- Inclusion of surgical periodontal therapy.
- Use of additional systemic or local adjunctive agents such as antibiotics, probiotics, or bisphosphonates in combination with Omega-3 therapy.
- Association of Omega-3 supplementation with other adjunctive approaches, including laser therapy or antimicrobial photodynamic therapy.
- Studies not reporting quantitative clinical outcomes, not meeting PICO eligibility, or lacking sufficient methodological detail.

### 2.6 Quality assessment

Two reviewers (GB, FT) independently evaluated all included studies for relevance, eligibility, and risk of bias using the Cochrane Collaboration's Risk of Bias 2 (RoB 2) tool (36). Any disagreements between reviewers were resolved through discussion and consensus with a third investigator (MGL).

### 2.7 Data extraction and collection process

Following the screening process, two authors (LM and MGL) independently extracted data from the included studies using standardized data extraction forms. Discrepancies were resolved through discussion with a third reviewer (EM) until consensus was reached. Extracted data included bibliographic information, study design, participant characteristics, intervention details (dosage, duration, and type of supplementation), follow-up period, and clinical outcomes.

The primary outcomes were changes in CAL and PPD, reported as mean and standard deviation (SD) for both test and control groups. When available, short-term outcomes (3 and/or 6 months) were extracted for meta-analysis using Review Manager software (RevMan, version 9.14.0) (37).

### 2.8 Statistical analysis

Data from the included studies were organized into evidence tables and summarized descriptively to identify variations in study design, participant characteristics, intervention protocols, and reported outcomes. Agreement between reviewers during study selection and data extraction was assessed using Cohen's kappa coefficient (κ).

For continuous outcomes, CAL gain and PPD reduction, mean differences and corresponding SD were extracted or calculated from the included studies. When sufficient data were available, meta-analyses were performed using the weighted mean difference (WMD) and 95% confidence intervals (CI). Short-term outcomes (3 and/or 6 months) were considered to ensure comparability across studies. Random-effects or fixed-effects models were applied according to the degree of statistical heterogeneity, which was assessed using Cochrane's *Q*-test (*p* < 0.05) and quantified by the *I*^2^ statistic. *I*^2^ values below 50% were interpreted as low heterogeneity, whereas those above 75% indicated substantial heterogeneity. Forest plots were generated to visualize the individual and pooled effects. Given the inclusion of studies involving systemically healthy (with or without ASA), diabetic populations and postmenopausal women, subgroup analyses were conducted to explore the potential influence of systemic conditions on treatment outcomes. Publication bias was evaluated using funnel plots and Egger's test for small-study effects (38).

All statistical analyses were performed using Review Manager (RevMan, version 9.14.0; Cochrane Collaboration, Oxford, UK) (37).

### 2.9 Assessment of result certainty

The certainty of the evidence was assessed using the Grading of Recommendations, Assessment, Development, and Evaluation (GRADE) approach (39). The GRADE assessment considered five domains: risk of bias, imprecision, inconsistency, indirectness, and publication bias, which were evaluated using the GRADEpro GDT software (40).

## 3 Results

### 3.1 Search results and selection of included studies

**Figure 1:**
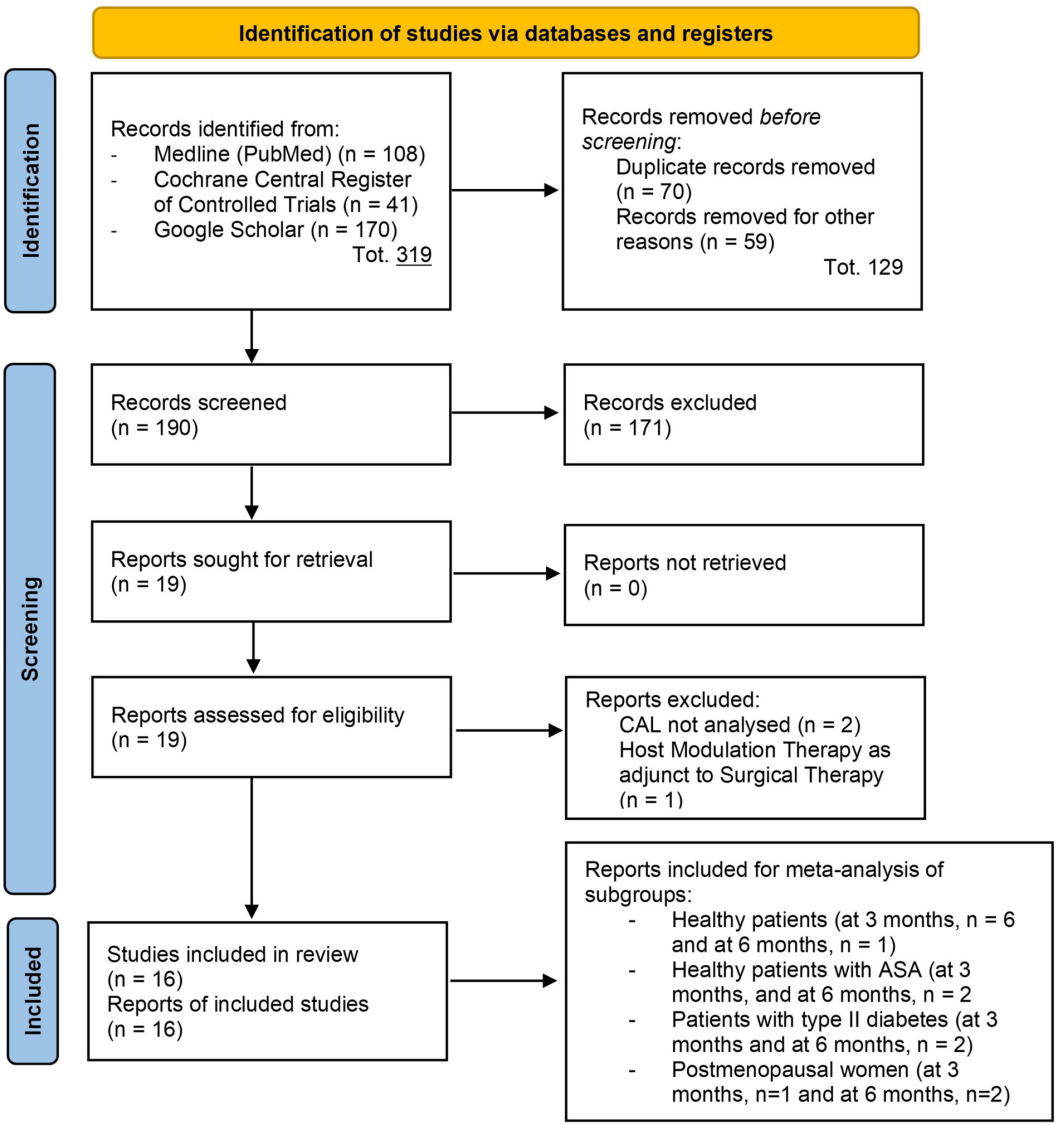
PRISMA 2020 flow diagram illustrating the study identification, screening, eligibility, and inclusion process for the systematic review.

A total of 319 references were identified through the electronic search conducted across MEDLINE (PubMed), the Cochrane Central Register of Controlled Trials, and Google Scholar. After creating a single list and removing 129 duplicate records, 190 studies remained for title and abstract screening. Independent screening resulted in the exclusion of 171 articles, with a high inter-reviewer agreement (*κ* = 0.91). Consequently, 19 articles were retrieved and assessed in full text. After full-text evaluation, two studies were excluded for not reporting CAL data (41, 42), and one was excluded for investigating Omega-3 PUFA as an adjunct to surgical therapy instead of NSPT (43). The remaining 16 studies met the inclusion criteria and were included in the qualitative synthesis. Of these, nine studies provided sufficient quantitative data and were included in the meta-analysis. Details of the search process are provided in Figure 1 (32).

### 3.2 Characteristics of included studies

#### 3.2.1 Study design

All the included studies were parallel-design, single center, RCTs published between 2010 and 2025 (44–59). Thirteen studies adopted a double-blind design, two were single-blind (50, 56), and in one, the blinding protocol was not clearly stated (55). All studies were conducted in a single center. Details are provided in Table 1.

**Table 1:** Characteristics of included studies.

Author (year)	Country	Study design	Participants (Sample/Diagnosis)	DoS/Follow-up (months)	Interventions	Outcomes	ASA	Key findings	RoB (overall)
Castro Dos Santos et al. (2020)	Brazil	RCT, parallel-arm, double-blind, single center.	75 analyzed; ≥35 years; type 2 diabetes; moderate-to-severe generalized chronic periodontitis	2/6	CG: periodontal debridement + placebo; TG1: NSPT + Omega-3 PUFA (3 g fish oil/day) + ASA 100 mg/day for 2 months after debridement; TG2: same supplementation for 2 months before debridement	CAL, PPD, GR, BoP, PI, glycemic control	Yes (not in CG)	Supplementation with Omega-3 PUFA and ASA significantly improved CAL and PPD compared with placebo, particularly when administered after periodontal debridement.	Low
Deore et al. (2014)	India	RCT, parallel-arm, double-blind, single center.	58 analyzed; 30–60 years; moderate/severe chronic periodontitis	3/3	CG: SRP + placebo (300 mg liquid paraffin once daily, 12 wks); TG: SRP + Omega-3 PUFA 300 mg/day (180 mg EPA + 120 mg DHA) for 12 wks	CAL, PPD, GI, PI, BoP	No	Adjunctive Omega-3 PUFA with SRP led to greater reductions in CAL and PPD compared to SRP alone, indicating modulation of the host inflammatory response.	Low
Elgendy et al. (2018)	Egypt	RCT, parallel-arm, double-blind, single center.	50 postmenopausal women (45–60 years) with generalized chronic periodontitis	6/6	CG: SRP + placebo (olive-oil capsules twice daily); TG: SRP + Omega-3 PUFA 1,000 mg twice daily (200 mg DHA+ 300 mg EPA per capsule)	CAL, PPD, GI, PI, GCF samples	No	ω-3 PUFA supplementation in postmenopausal women produced significant improvements in CAL and PPD versus placebo after 6 months.	Some concerns
El-Sharkawy et al. (2010)	Egypt	RCT, parallel-arm, double-blind, single center.	80 analyzed; 30–70 years; systemically healthy; untreated advanced chronic periodontitis	6/6	CG: SRP + placebo; TG: SRP + Omega-3 PUFA (3,000 mg fish oil/day) + ASA 81 mg/day	PI, GI, BoP, CAL, PPD, serum and GCF inflammatory markers	Yes (not in CG)	Combined Omega-3 PUFA and low-dose ASA enhanced CAL gain and reduced gingival inflammation, supporting inflammation-resolution–based therapy.	Low
Elwakeel et al. (2015)	Egypt	RCT, parallel-arm, double-blind, single center.	40 analyzed; type 2 diabetes >2 years (HbA1c 7%–8%); moderate–severe periodontitis	6/6	CG: SRP + placebo (coconut oil) + ASA (lactose tablet); TG: SRP + Omega-3 PUFA 1,000 mg three times/day + ASA 75 mg/day	PI, GI, CAL, PPD, glycemic control, GCF cytokines	Yes	Combined Omega-3 PUFA and ASA therapy improved CAL and metabolic control in diabetic patients with chronic periodontitis.	Low
Keskiner et al. (2017)	Turkey	RCT, parallel-arm, double-blind, single center.	30 analyzed (16M/14F); mean age ≈ 41–43 years; systemically healthy with chronic periodontitis	6/6	CG: SRP + placebo; TG: SRP + Omega-3 PUFA (6.25 mg EPA + 19.19 mg DHA)	PI, GI, BoP, CAL, PPD, salivary TNF-α	No	Omega-3 PUFA supplementation did not significantly affect clinical parameters but reduced salivary TNF-α levels, suggesting partial host-response modulation.	Some concerns
Kujur et al. (2020)	India	RCT, parallel-arm, single-blind, single center.	76 analyzed; Age range: 30–70 years; untreated chronic generalized periodontitis	1/3	CG: SRP alone; TG: SRP + Omega-3 PUFA 500 mg/day for 1 month (EPA 180 mg/DHA 120 mg)	CAL, PPD, GI, PI	No	Short-term Omega-3 PUFA supplementation improved CAL and PPD in patients with chronic periodontitis compared to SRP alone.	Some concerns
Martinez et al. (2013)	Brazil	RCT, parallel-arm, double-blind, single center.	21 analyzed; mean age 46.0 ± 8.8 years; generalized chronic periodontitis	4/4	CG: SRP + placebo (3 caps/day, 4 months); TG: SRP + Omega-3 PUFA (3 caps/day, total 900 mg EPA + DHA/day)	Serum long-chain PUFA levels, CAL, PPD, BoP, PI	No	No statistically significant improvement in clinical or biochemical parameters was observed following Omega-3 PUFA supplementation.	Some concerns
Martinez et al. (2014)	Brazil	RCT, parallel-arm, double-blind, single center.	15 analyzed (6M/9F); ≈ 46 years; generalized chronic periodontitis	12/12	CG: SRP + placebo, 3 caps/day; TG: SRP + Omega-3 PUFA, 3 caps/day (300 mg Omega-3; 180 mg EPA + 120 mg DHA)	BoP, PI, CAL, PPD, serum lipid profile	No	Long-term Omega-3 PUFA supplementation (12 months) did not result in additional CAL gain or PPD reduction compared with placebo.	Some concerns
Maybodi et al. (2022)	Iran	RCT, parallel-arm, double-blind, single center.	30 analyzed; Age range: 30–70 years; periodontitis Stage II–IV, Grade B	3/3	CG: SRP + placebo (soybean-oil soft gels); TG: SRP + Omega-3 PUFA fish-oil soft gels 1,000 mg/day (300 mg Omega-3; 180 mg EPA + 120 mg DHA)	CAL, PPD, BI	No	Adjunctive Omega-3 PUFA significantly improved clinical outcomes (CAL and PPD) compared with soybean-oil placebo in Stage II–IV periodontitis.	Low
Naqvi et al. (2017)	USA (Massachusetts)	RCT, parallel-arm, double-blind, single center.	46 analyzed; ≥40 years; moderate periodontitis	3/3	CG: SRP + placebo [950 mg (50% corn oil/50% soybean oil)] + ASA 81 mg/day; TG: SRP + DHA (4 caps/day, 500 mg oil each) + ASA 81 mg/day	PPD, BoP, CAL, GI, PI	Yes	Co-administration of DHA and ASA reduced PPD and gingival inflammation and modulated the subgingival microbiota composition.	Some concerns
Rampally et al. (2019)	India	RCT, parallel-arm, single center.	42 analyzed; 30–65 years; type 2 diabetes (HbA1c ≥ 6.5%)	3/3	CG: SRP + placebo (empty gelatin caps); TG1: SRP + ASA 75 mg/day; TG2: SRP + Omega-3 PUFA 500 mg twice/day for 3 months	GI, CAL, PPD, PTX3 levels	Yes (TG1)	Both ASA and Omega-3 PUFA reduced inflammatory marker (PTX3) levels; ω-3 PUFA showed greater anti-inflammatory efficacy in diabetic patients.	High
Stańdo et al. (2020)	Poland	RCT, parallel-arm, single-blind, single center.	30 analyzed; 22–70 years; generalized Stage III–IV periodontitis	3/3	CG: SRP; TG: SRP + Omega-3 PUFA 10 ml twice/day (≈2.6 g EPA + 1.8 g DHA + other marine lipids)	CAL, PPD, GI, FMPI, GR, BoP, saliva sampling	No	High-dose Omega-3 PUFA supplementation improved CAL and PPD after 3 months, supporting its use as an adjunct to NSPT.	Some concerns
Stańdo-Retecka et al. (2023)	Poland	RCT, parallel-arm, double-blind, single center.	40 analyzed; 22–70 years; generalized Stage III–IV periodontitis	6/6	CG: SRP; TG: SRP + Omega-3 PUFA 10 ml twice/day (same formulation as Stańdo 2020)	CAL, PPD, FMPI, GR, microbiological outcomes	No	Omega-3 PUFA led to significant improvements at 3 months, though effects diminished by 6 months; no microbiological differences observed long-term.	Some concerns
Eldessouky et al. (2024)	Egypt	RCT, parallel-arm, double-blind, single center.	20 postmenopausal women (45–55 years) with periodontitis	12/12	CG: SRP + PLACEBO (soft gelatinous capsule containing olive oil); TG: SRP + Omega-3 PUFA 1,000 mg	PI, GI, PPD, CAL, CGF samples (osteocalcin level)	No	The study's significant reduction in GCF AST levels suggests potential mitigation of tissue damage and inflammation after periodontal therapy augmented by Omega-3 FAs.	Some concerns
Araujo et al. (2025)	Brazil	RCT, parallel-arm, double-blind, single center.	38 analyzed; 18–35 years; generalized Stage III–IV Grade C periodontitis (former generalized aggressive)	6/6	CG: FMUD + placebo (3 caps/day for 180 days); TG: FMUD + Omega-3 900 mg/day + ASA 100 mg/day for 180 days	CAL, PPD, BoP, GR, GCF cytokines	Yes	Combined Omega-3 PUFA and ASA supplementation did not enhance clinical outcomes beyond NSPT alone, though immunoregulatory effects were noted.	Low

DoS, duration of supplementation; CG, control group; TG (/TG1/TG2), test group(s); ASA, acetylsalicylic acid; PUFA, polyunsaturated fatty acids; SRP, scaling and root planing; NSPT, non-surgical periodontal therapy; FMUD, full-mouth ultrasonic debridement; FMPI, full mouth plaque index; GCF, gingival crevicolar fluid; EPA, eicosapentaenoic acid; DHA, docosahexaenoic acid; CAL, clinical attachment level; PPD, probing pocket depth; GR, gingival recession; BoP, bleeding on probing; BI, bleeding index; PI, plaque index; GI, gingival index; TNF-α, tumor necrosis factor alpha; PTX3, pentraxin 3; RoB, risk of bias.

#### 3.2.2 Studies’ samples

Sample sizes ranged from 15 to 80 participants, all aged ≥18 years, except for Elwakeel (48), which did not specify participant age. All studies included patients diagnosed with periodontitis staged between Stage II and Stage IV, according to the current classification.

Three studies specifically enrolled patients with type 2 diabetes (44, 48, 55), while two included postmenopausal women (46, 58). One RCT focused exclusively on patients with generalized Stage III–IV Grade C periodontitis (59).

#### 3.2.3 Intervention/comparison

All included studies evaluated non-surgical periodontal therapy (NSPT) with subgingival instrumentation as the base treatment. The test groups received omega-3 polyunsaturated fatty acids (PUFA) supplementation, either alone or in combination with acetylsalicylic acid (ASA). Five studies evaluated the combined administration of omega-3 PUFA and ASA (44, 47, 48, 54, 59). Ten studies assessed omega-3 PUFA alone (45, 46, 49–53, 56–58), while one compared omega-3 and low-dose ASA as separate adjunctive regimens (55). Control groups received either NSPT alone or NSPT + placebo. Supplementation duration varied between 1 and 12 months, most commonly lasting 3 or 6 months.

#### 3.2.4 Follow-up period

Follow-up duration varied across studies: six trials had a 3-month follow-up (45, 50, 53–56), one had a 4-month follow-up (52), seven extended to 6 months (44, 46–49, 57, 59), and two had a 12-month follow-up (51, 58).

#### 3.2.5 Outcomes

Across all included studies, the primary outcomes were the clinical parameters CAL and PPD**,** reported as mean change (±SD) from baseline at short-term follow-ups (3–6 months). These variables were consistently used to assess the clinical efficacy of omega-3 PUFA supplementation, with or without acetylsalicylic acid (ASA), as an adjunct to NSPT. Baseline and end-of-follow-up values for trials with two study arms are summarized in Table 2, while studies with more than two arms are presented in Table 3. Two studies did not report the raw data; the authors of the present systematic review attempted to obtain them but received no response from the original authors (51, 52).

**Table 2:** Mean values of PPD and CAL at baseline and at the final evaluation for control and test groups across the included studies (single test group design).

Main Author (year)	Observation Period (months)	PPD (mm) ± SD				CAL (mm) ± SD			
		Baseline		Follow-up		Baseline		Follow-up	
		CG	TG	CG	TG	CG	TG	CG	TG
Deore et al. (2014)	3	4.05 ± 1.03	4.26 ± 1.10	2.77 ± 0.47	2.15 ± 0.53	5.20 ± 0.90	5.53 ± 0.95	3.72 ± 0.62	2.73 ± 0.98
Elgendy et al. (2018)	6	5.84 ± 0.61	6.00 ± 0.59	4.29 ± 0.75	3.46 ± 0.49	5.79 ± 0.72	5.96 ± 0.61	4.06 ± 0.59	3.40 ± 0.50
El-Sharkawy et al. (2010)	6	4.4 ± 0.7	4.2 ± 0.9	3.0 ± 1.0	2.2 ± 0.8	4.7 ± 1.0	4.5 ± 1.0	3.5 ± 1.3	2.5 ± 1.1
Elwakeel et al. (2015)	6	5.85 ± 0.99	5.7 ± 0.8	3.8 ± 0,7	2.8 ± 0.52	6.6 ± 1.19	6.0 5 ± 1.76	4.2 ± 0.52	3 ± 1.78
Keskiner et al. (2017)	6	3.73 (2.43–4.25)^a^	3.72 (2.23–4.75)^a^	2.38 (2.04–3.23)^a^	2.46 (1.83–3.32)^a^	4.20 (2.73–5.33)^a^	4.59 (3.04–5.31)^a^	3.10 (2.68–4.16)^a^	3.53 (2.42–4.08)^a^
Kujur et al. (2020)	3	3.73 ± 0.30	3.77 ± 0.33	3.23 ± 0.30	2.77 ± 0.33	4.62 ± 0.37	4.78 ± 0.25	4.10 ± 0.37	3.78 ± 0.25
Maybodi et al. (2022)	3	4.50 ± 1.01	5.06 ± 0.98	3.34 ± 0.94	2.65 ± 0.84	4.66 ± 1.01	5.24 ± 1.04	3.57 ± 0.92	3.07 ± 0.61
Naqvi et al. (2017)	3	2.6 ± 0.4	2.5 ± 0.3	2.6 ± 0.5	2.5 ± 0.2	2.5 ± 0.7	2.4 ± 0.7	2.5 ± 0.6	2.4 ± 0.7
Stańdo et al. (2020)	3	5.1 ± 0.8	5.0 ± 0.5	4.0 ± 0.7	3.7 ± 0.7	6.1 ± 1.1	5.8 ± 0.8	5.3 ± 1.0	4.4 ± 1.1
Stańdo-Retecka et al. (2023)	6	5.02 ± 0.71	5.01 ± 0.49	3.64 ± 0.82	3.49 ± 0.80	5.87 ± 1.02	5.71 ± 0.79	4.77 ± 1.02	4.25 ± 1.29
Eldessouky et al. (2024)	12	5.11 ± 0.54	5.18 ± 0.65	4.89 ± 0.63	3.33 ± 0.34	5.31 ± 0.55	5.29 ± 0.37	5.22 ± 0.65	2.99 ± 0.43
Araujo et al. (2025)	6	3.88 ± 0.15	3.74 ± 0.15	3.37 ± 0.14	3.25 ± 0.13	3.94 ± 0.17	4.01 ± 0.21	3.59 ± 0.17	3.64 ± 0.2

PPD, probing pocket depth; CAL, clinical attachment loss; CG, control group; TG (/TG1/TG2), test group.

a Median (25–75 percentiles).

**Table 3:** Mean values of PPD and CAL at baseline and at the final evaluation for control and test groups across the included studies (multiple test group design).

Main Author (Year)	Observation Period (months)	PPD (mm) ± SD						CAL (mm) ± SD					
		Baseline			Follow-up			Baseline			Follow-up		
		CG	TG1	TG2	CG	TG1	TG2	CG	TG1	TG2	CG	TG1	TG2
Castro Dos Santos et al. (2020)	6	3.3 ± 0.5	3.3 ± 0.6	3.2 ± 0.5	2.9 ± 0.4	2.9 ± 0.4	2.9 ± 0.4	3.8 ± 0.6	3.9 ± 0.8	3.8 ± 0.8	3.6 ± 0.6	3.4 ± 0.8	3.5 ± 0.8
Rampally et al. (2019)	3	6.43 ± 0.51	6.43 ± 0.51	6.71 ± 0.47	4.43 ± 0.51	4.43 ± 0.51	4.71 ± 0.47	5.43 ± 0.51	5.43 ± 0.51	5.71 ± 0.47	3.43 ± 0.51	3.43 ± 0.51	3.71 ± 0.47

PPD, probing pocket depth; CAL, clinical attachment loss; CG, control group; TG (/TG1/TG2), test group.

Secondary outcomes varied across studies but included additional clinical, biochemical, and microbiological parameters.

- Clinical outcomes: BoP and PI were commonly evaluated as indicators of gingival inflammation (46, 53, 56, 58).
- Biochemical and immunological markers**:** several studies measured inflammatory mediators such as TNF-α, IL-1β, and C-reactive protein (47–49), serum fatty acid profiles (51, 52) or GCF osteocalcin level (58).
- Microbiological outcomes**:** shifts in subgingival microbial composition were assessed in selected trials (54, 57).
- Patient-reported outcome measures (PROMs)**:** tolerability and compliance with ω-3 supplementation were also reported in several studies, with no adverse effects observed.

Collectively, the heterogeneity of secondary outcomes reflects differences in study aims and methodologies but supports the biological rationale for omega-3 PUFA supplementation as a host-modulatory strategy in periodontitis management.

#### 3.2.6 Risk of bias assessment

**Figure 2:**
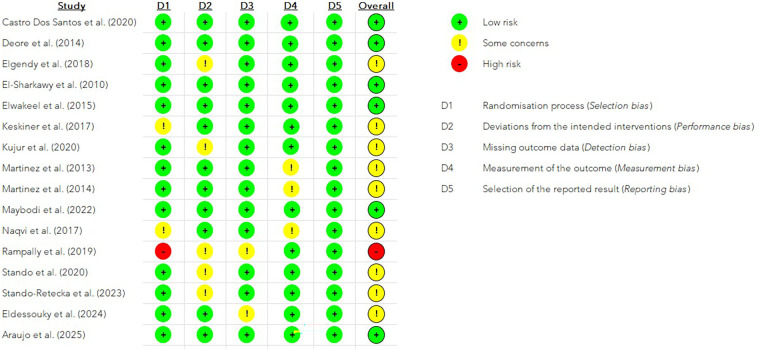
Risk of bias assessment of the included randomized controlled trials using the cochrane RoB 2 tool, presented as a traffic light plot.

Detailed risk of bias assessment is presented in Figure 2. Overall, most studies exhibited a low to moderate (“some concerns”) risk of bias according to the Cochrane RoB 2 tool. One study showed a high risk of bias due to inadequate randomization (55), while nine presented moderate concerns mainly related to blinding or incomplete data reporting (46, 49–52, 54, 56–58). The remaining trials were judged to have a low overall risk of bias (44, 45, 47, 48, 53, 59).

#### 3.2.7 Publication bias and assessment of result certainty

Publication bias was evaluated for the primary outcomes at 3- and 6-month follow-ups. Visual inspection of the funnel plots revealed an asymmetrical distribution of studies around the pooled effect size in three of the comparisons (see Supplementary Figures S2, S3, S5), suggesting a low risk of publication bias, whereas one plot showed a symmetrical distribution (Supplementary Figure S4), indicating no evidence of publication bias.

In the analysis of PPD reduction at 3, the plot was largely symmetrical and centered on the overall mean difference, indicating that small-study effects were unlikely to have influenced the pooled estimates. Due to the limited number of studies included in each analysis (<10), Egger's test for small-study effects was not performed, in accordance with Cochrane methodological standards (31).

### 3.3 Meta-analysis

#### 3.3.1 CAL changes

Meta-analysis was conducted on studies reporting CAL changes at 3- and 6-month follow-ups, stratified by patient condition (healthy with or without ASA, with type II diabetes or postmenopausal women).

At 3 months, the pooled analysis revealed a mean difference (MD) of −0.49 mm [95% CI −0.75, −0.23; *p* = 0.0002], favoring SRP + omega-3 PUFA (±ASA) over SRP alone, with high heterogeneity (*I*^2^ = 84%).

**Figure 3:**
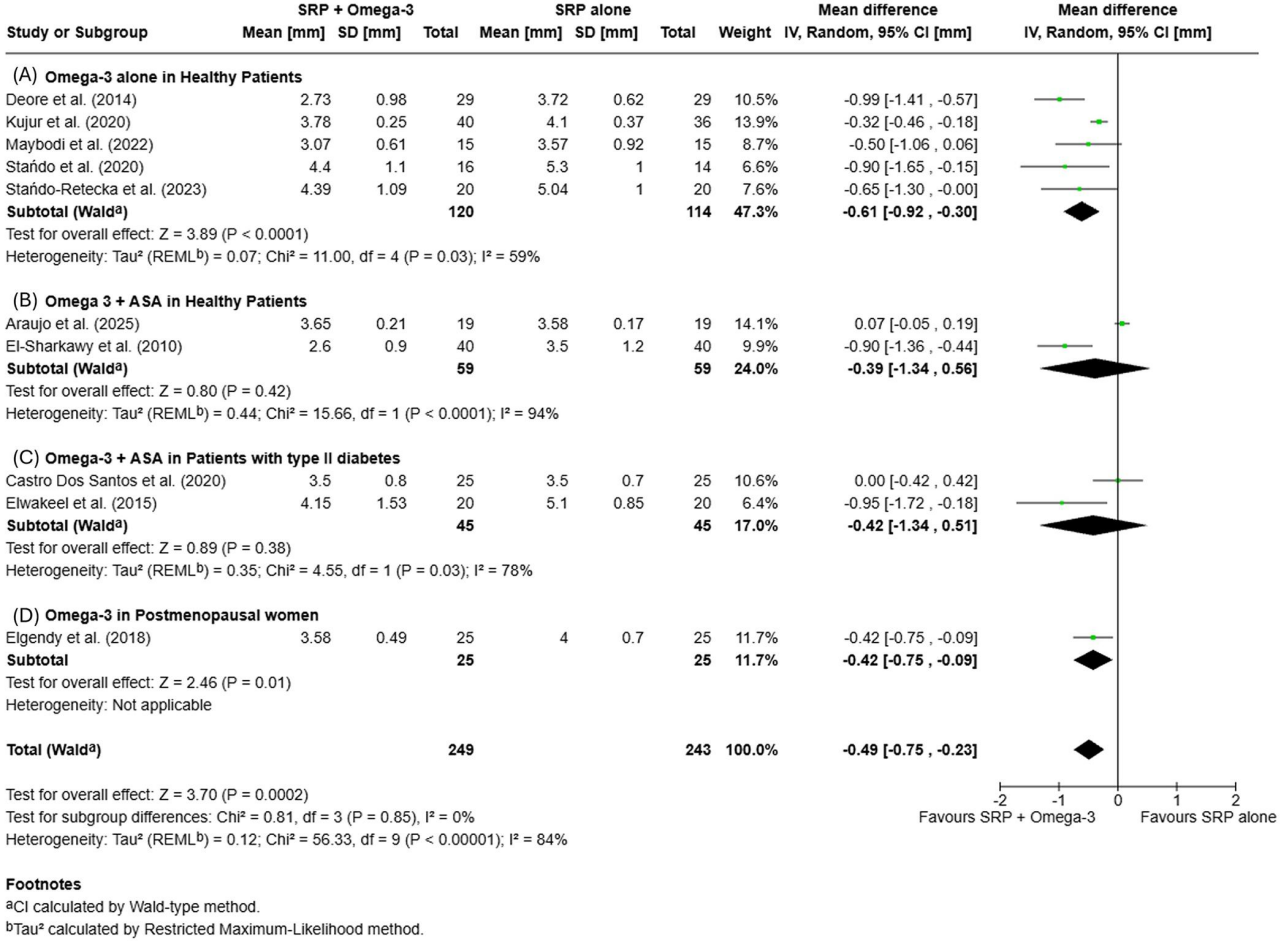
Forest plots of mean clinical attachment level (CAL) changes at 3 months: **(A)** Omega-3 supplementation alone in systemically healthy patients; **(B)** Omega-3+ acetylsalicylic acid (ASA) in systemically healthy patients; **(C)** Omega-3+ ASA in patients with type II diabetes; **(D)** Omega-3 supplementation in postmenopausal women.

**Figure 4:**
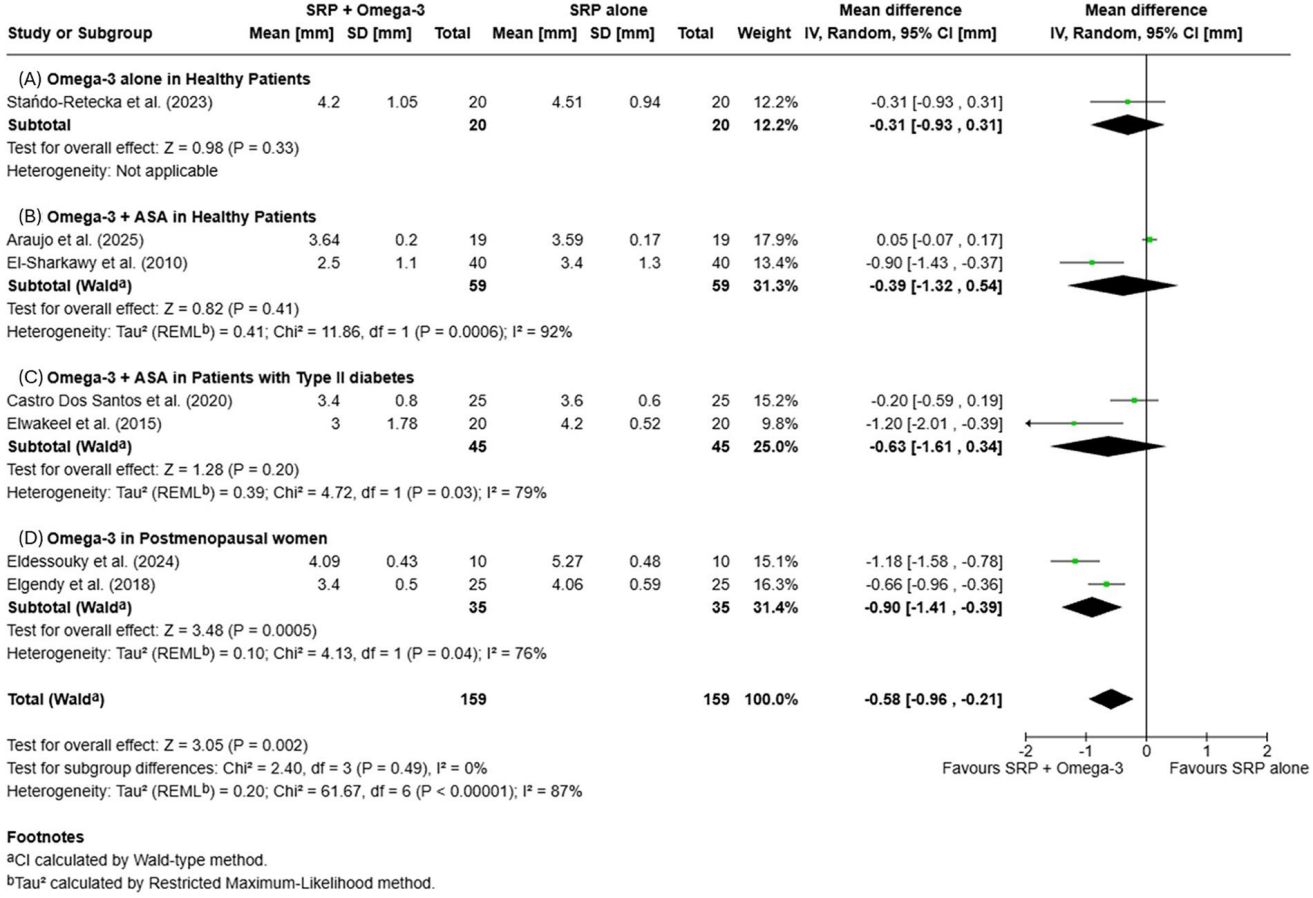
Forest plots of mean clinical attachment level (CAL) changes at 6 months: **(A)** Omega-3 supplementation alone in systemically healthy patients; **(B)** Omega-3+ acetylsalicylic acid (ASA) in systemically healthy patients; **(C)** Omega-3+ ASA in patients with type II diabetes; **(D)** Omega-3 supplementation in postmenopausal women.

At 6 months, the difference remained in favor of the test group [MD = −0.58 mm (95% CI −0.96, −0.21); *p* = 0.002], statistically significant, with high heterogeneity (*I*^2^ = 87%). Subgroup analysis did not reveal significant differences between subgroups. Details of pooled CAL changes are presented in Figures 3A–D, 4A–D.

#### 3.3.2 PPD reduction

**Figure 5:**
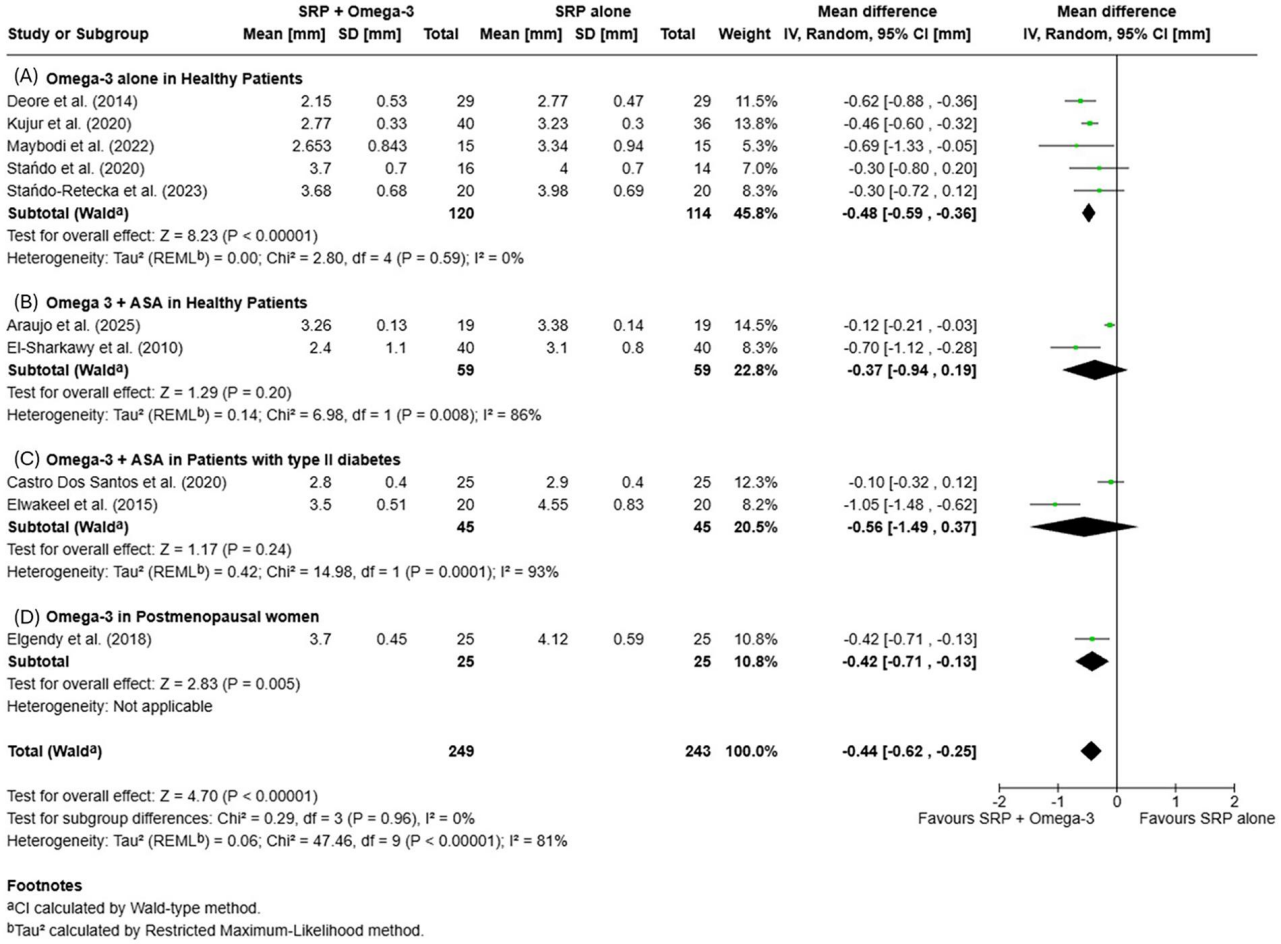
Forest plots of mean probing pocket depth (PPD) changes at 3 months: **(A)** Omega-3 supplementation alone in systemically healthy patients; **(B)** Omega-3+ acetylsalicylic acid (ASA) in systemically healthy patients; **(C)** Omega-3+ ASA in patients with type II diabetes; **(D)** Omega-3 supplementation in postmenopausal women.

**Figure 6:**
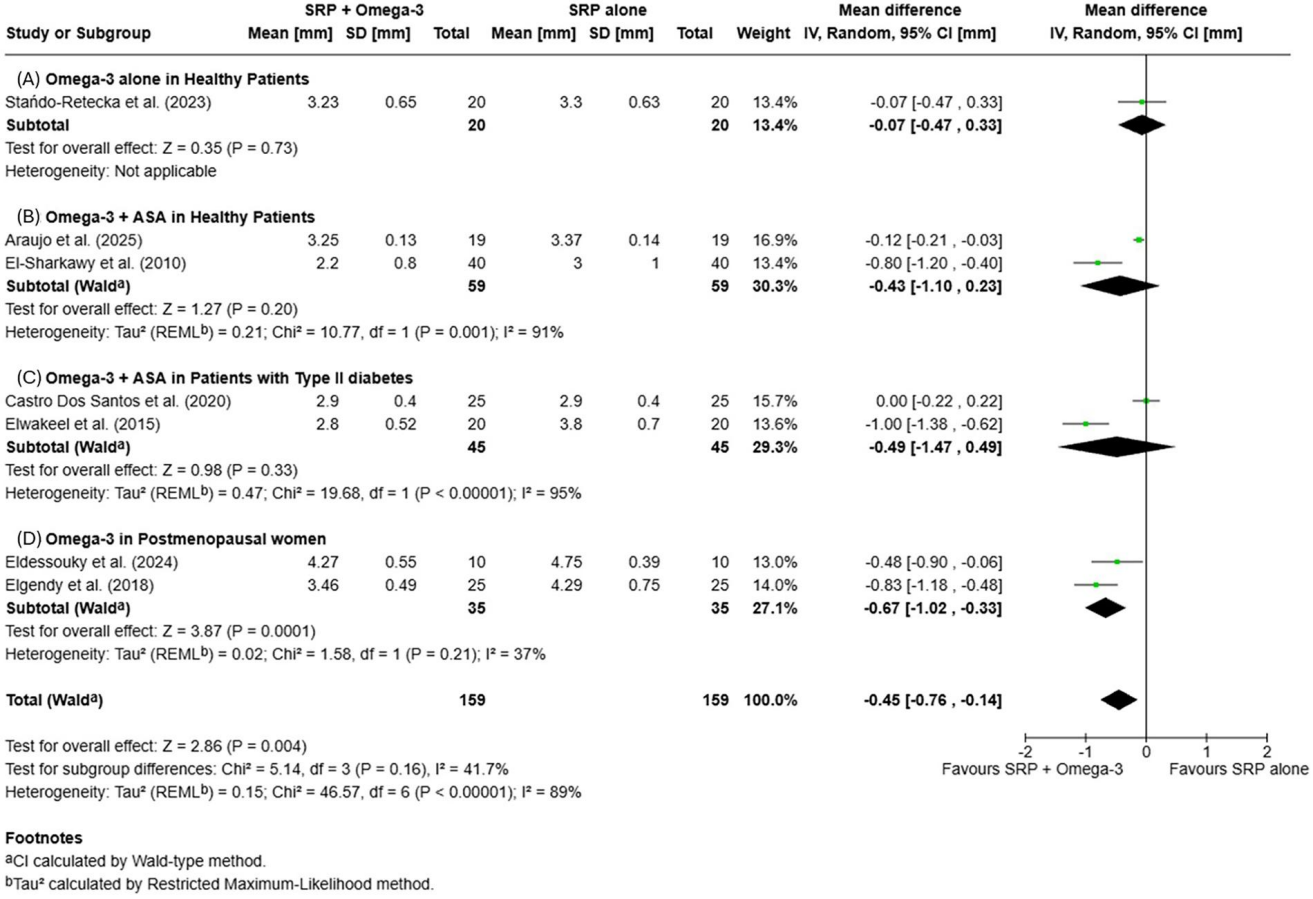
Forest plots of mean probing pocket depth (PPD) changes at 6 months: **(A)** Omega-3 supplementation alone in systemically healthy patients; **(B)** Omega-3+ acetylsalicylic acid (ASA) in systemically healthy patients; **(C)** Omega-3+ ASA in patients with type II diabetes; **(D)** Omega-3 supplementation in postmenopausal women.

PPD reduction was assessed across studies reporting outcomes at 3- and 6-month follow-ups. At 3 months, a statistically significant additional PPD reduction was observed in the SRP + omega-3 PUFA (±ASA) group compared with SRP alone [MD = −0.44 mm (95% CI −0.62, −0.25); *p* < 0.00001], with high heterogeneity (I^2^ = 81%). At 6 months, the mean difference remained favorable and statistically significant [MD = −0.45 mm (95% CI −0.76, −0.14); *p* = 0.004; *I*^2^ = 89%]. Subgroup comparisons showed no statistically significant differences. Forest plots summarizing PPD outcomes are displayed in Figures 5A–D, 6A–D.

## 4 Discussion

The present systematic review and meta-analysis demonstrated that adjunctive supplementation with Omega-3 PUFA, with or without ASA, provided significant short-term clinical benefits when combined with NSPT. The quantitative synthesis revealed a statistically significant gain in CAL and reduction in PPD at 3 months, while 6-month outcomes showed a favorable but non-significant trend. No adverse effects were reported across the included trials. These findings suggest that Omega-3 PUFA supplementation may enhance the initial healing response following subgingival instrumentation, likely through its host-modulating and pro-resolving properties.

Regarding the study hypothesis, the pooled 3-month and 6-month analyses for both CAL and PPD demonstrated a statistically significant advantage of NSPT supplemented with omega-3 PUFA (±ASA) over NSPT alone. Therefore, the null hypothesis stating that omega-3 supplementation does not provide additional clinical benefits compared with subgingival instrumentation alone can be rejected for short-term follow-ups. However, further studies are required with longer observation period. Although the magnitude of clinical improvement was modest, the consistency of the effect across diverse populations, including both systemically healthy and diabetic patients, supports the biological plausibility of Omega-3 PUFA as an adjunctive host-modulatory approach.

The findings of the present review are broadly consistent with the three most recent SRs and MAs on the adjunctive use of omega-3 in NSPT. Van Ravensteijn et al. reported an additional PPD reduction of 0.39 mm and a CAL gain of 0.41 mm in favour of SRP + omega-3, interpreting this effect as moderate but clinically plausible (27). In the present SR, pooled estimates at 3 months (PPD ≈ 0.4–0.5 mm; CAL ≈ 0.5 mm) fall in the same range, confirming that the magnitude of benefit is small-to-moderate and comparable across different populations and dosing regimens. Similarly, Dos Santos et al. found that Omega-3 supplementation as an adjunct to NSPT produced statistically significant improvements in both PPD and CAL, but emphasized the heterogeneity in dose, duration and association with ASA and concluded that the overall certainty of evidence was “low to moderate” (25). The present systematic review, which includes three additional and more recent RCTs (57–59), confirms this picture: the effect is significant at both 3 and 6 months, while heterogeneity remains high, particularly when studies involving diabetic patients or Omega-3+ ASA protocols are pooled. Chatterjee et al. also reported significant improvements in CAL and PPD, but, differently from our review, did not systematically separate healthy and systemically compromised subjects, and most of the included RCTs had short follow-up (≤3 months) (26). By stratifying outcomes by health status, ASA addiction and time point, our analysis shows that the signal is strong in the short term and that part of the variability seen in previous MAs is likely driven by mixed populations and non-comparable control groups.

Although these results align with previous evidence suggesting a modest yet consistent clinical benefit, the EFP guidelines currently consider Omega-3 PUFA supplementation as an emerging host-modulatory approach rather than a standard therapeutic adjunct (24). The EFP consensus emphasizes that evidence remains insufficient to justify routine recommendation, pending further RCTs with standardized dosages and longer follow-ups (24). The present findings strengthen this position, suggesting that Omega-3 supplementation may indeed provide measurable clinical benefits, although its integration into clinical guidelines should await confirmation from larger, methodologically robust studies.

Beyond clinical parameters, several studies included in this review investigated biochemical, microbiological, and patient-reported outcomes, providing mechanistic insights into the potential adjunctive role of Omega-3 (44, 46–49, 51, 52, 55–59). The overall pattern of these findings is in line with previous systematic reviews, which consistently reported anti-inflammatory modulation and microbial profile shifts associated with Omega-3 supplementation. Across the included RCTs, reductions in systemic and local inflammatory mediators such as IL-1β, TNF-α, and CRP were observed in the Omega-3 groups (47–49). The downregulation of these cytokines is biologically plausible given that EPA and DHA are precursors of specialized pro-resolving mediators (SPMs) such as resolvins and protectins, which promote resolution of inflammation rather than mere suppression. Interestingly, Araujo also reported upregulation of immunoregulatory mediators despite the absence of significant clinical changes, supporting the hypothesis that biochemical modulation may precede measurable periodontal improvements (59). Selected studies reported beneficial shifts in subgingival microbiota, with reduced abundance of *Porphyromonas gingivalis* and *Tannerella forsythia* and partial restoration of symbiotic bacterial communities (54, 57). These findings parallel those of two systematic reviews, suggesting that Omega-3 may influence microbial ecology indirectly through modulation of the host inflammatory environment rather than through direct antibacterial effects (26, 29). Most trials reported high compliance and absence of adverse effects (46, 49, 53, 56, 57). One study specified that thirteen subjects in the intervention group experienced side effects such as nausea, abdominal discomfort, and fish-scented halitosis; however, these adverse effects were mild and did not lead to discontinuation of the treatment (48). One study only specified that there were no adverse events or presented complications due to long-term use of low-dose ASA (44). The only recurrent negative report were the unpleasant taste of the capsules or nausea, consistent with previous reviews. Overall, the tolerability profile of omega-3 remains favorable, supporting its potential as a safe adjunctive therapy.

The combination of Omega-3 supplementation with low-dose ASA is biochemically supported by evidence that aspirin activates an additional synthetic pathway for lipoxins. By acetylating cyclooxygenase-2 (COX-2), aspirin modifies its enzymatic activity, promoting the formation of 15-R-HETE, which is then converted by 5-LOX into aspirin-triggered lipoxins (ATLs), such as 15-epi-LXA_4_ and 15-epi-LXB_4_. These mediators enhance the endogenous resolution of inflammation, acting synergistically with omega-3–derived resolvins and protectins (15, 17, 23, 60). Among the studies included in this review, six RCTs evaluated the adjunctive use of ASA (44, 47, 48, 54, 55, 59). In five of these, ASA was co-administered with Omega-3 (44, 47, 48, 54, 59), while one directly compared the two agents in separate test groups (55). Despite methodological heterogeneity and short follow-ups, these studies collectively suggest that the addition of ASA may potentiate anti-inflammatory effects through enhanced lipid mediator biosynthesis. However, this did not consistently translate into superior clinical improvements in CAL or PPD compared with Omega-3 alone. The dosages of ASA varied across trials (from 75 to 100 mg/day), which may have influenced comparability. Only two studies used the standard 100 mg dose, while the others adopted lower regimens (75–81 mg) (44, 59). Although the biochemical rationale for ASA as an amplifier of pro-resolving pathways is well established, its clinical relevance in NSPT remains uncertain and warrants further standardized trials.

A further source of variability across studies lies in the wide range of Omega-3 dosages and supplementation durations. Daily doses varied from 500 mg to 3,000 mg, with heterogeneous EPA:DHA ratios (most frequently 3:2 or 2:1) and administration periods ranging between 4 weeks and 6 months. These differences likely contributed to the variability in CAL and PPD improvements. Moreover, only a few studies verified compliance or measured plasma fatty acid levels, limiting the interpretation of the true systemic bioavailability of the supplement.

This systematic review and meta-analysis presents some limitations that should be acknowledged when interpreting the findings. The main limitation concerns the heterogeneity among studies, arising from variations in Omega-3 dosage, supplementation duration, follow-up length, and methodological design. These differences reduced the possibility of pooling all available data and likely contributed to the broad confidence intervals observed in the quantitative synthesis. Another limitation is the short duration of most follow-ups, generally ranging from 3 to 6 months, which does not allow a full evaluation of the long-term stability of the clinical improvements observed in CAL and PPD. The variability in formulations and composition of Omega-3 supplements (EPA:DHA ratio, capsule content, and concurrent use of ASA also complicates the interpretation of dose-response effects and prevents the identification of an optimal therapeutic protocol. In several trials, a moderate risk of bias was identified, mainly related to randomization, blinding, or incomplete reporting, which may influence the robustness of the pooled outcomes. Furthermore, secondary outcomes, such as inflammatory and microbiological parameters, were inconsistently evaluated and often based on small subgroups, limiting the strength of mechanistic conclusions.

Taken together, these methodological shortcomings inevitably affect the overall certainty of the available evidence. When evaluating the certainty of evidence using the GRADE approach (39) with GRADEpro GDT software (40), only one of the meta-analyses was rated as having *moderate* certainty, while the others were judged to have *low* certainty. This finding suggests caution in data interpretation and underscores the need for future randomized trials with improved methodological rigor to minimize potential sources of bias.

Although subgroup analyses were performed according to systemic condition, use of acetylsalicylic acid and follow-up duration, none of these factors fully explained the substantial heterogeneity observed, which likely reflects combined differences in Omega-3 dosage and formulation, supplementation timing and patient characteristics. Accordingly, a random-effects model was applied and studies at high risk of bias were excluded from the quantitative synthesis. Sensitivity analyses were considered but not performed, as no arbitrary methodological decisions or clear outliers were present and each comparison included a limited number of studies, in line with Cochrane methodological guidance.

Despite these limitations, the consistency of short-term improvements across studies supports the biological rationale for Omega-3 as a host-modulating adjunct in NSPT, while highlighting the need for well-designed, long-term trials to confirm its clinical relevance and to define optimal dosing strategies.

## 5 Conclusions

The present systematic review and meta-analysis indicates that Omega-3 PUFA, alone or in combination with low-dose ASA, may improve short-term clinical outcomes when used as an adjunct to NSPT. Significant benefits were observed in PPD reduction and CAL gain at both 3 and 6 months, with no reported adverse effects. Therefore, Omega-3 PUFA supplementation can be considered a safe, non-invasive, and accessible adjunctive strategy to enhance periodontal healing and host modulation, particularly in systemically compromised patients. According to the GRADE assessment, the certainty of evidence supporting these findings was low to moderate; therefore, the observed clinical benefits should be interpreted with caution when considering clinical implications. Despite encouraging short-term results, evidence remains limited by heterogeneity in dosage, treatment duration, and methodological design. Future randomized controlled trials should aim to standardize Omega-3 PUFA formulations and evaluate long-term outcomes beyond 6 months.

## Data Availability

The data analyzed in this study is subject to the following licenses/restrictions: the datasets analyzed in this study consist of data extracted from previously published randomized clinical trials. These original datasets are not publicly available, as they are owned by the respective study authors and subject to publication restrictions. Requests to access these datasets should be directed to gianluca.benincasa@student.univaq.it.
