# Supplementary material for: The role of omega-3 polyunsaturated fatty acids in the non-surgical management of periodontitis: a systematic review and meta-analysis
Source: Front Oral Health. 2026 Mar 13;7:1761032. doi: 10.3389/froh.2026.1761032 (PMC13021888; doi:10.3389/froh.2026.1761032)

Supplementary Material

# Supplementary Figures and Tables

**Table S2.** GRADE Summary of Findings – assessed with: GRADEpro Guideline Development Tool (GRADEpro GDT).

**PICO question:** In patients with stage II-IV periodontitis, does supplementation with omega-3 polyunsaturated fatty acids, with or without acetylsalicylic acid, in addition to non-surgical periodontal therapy, improve clinical outcomes compared with non-surgical therapy alone?

| **Certainty assessment** | | | | | | | **Number of Patients** | | **Effect** | | **Certainty** | **Summary of findings** |
| --- | --- | --- | --- | --- | --- | --- | --- | --- | --- | --- | --- | --- |
| **Number of studies** | **Study design** | **Risk of bias** | **Inconsistency** | **Indirectness** | **Imprecision** | **Other considerations** | **NSPT + Omega-3 PUFA (± ASA)** | **NSPT + Placebo** | **Relative effect (95% CI)** | **Absolute effect (95% CI)** |  |  |
| Clinical Attachment Loss at 3 months (follow-up: mean 3 months; assessed with: RevMan) | | | | | | | | | | | | |
| 10 | Randomized trials | serious^a^ | serious^b^ | not important | not important | Suspected publication bias^c^ | 249 | 243 | - | MD **0.49 mm lower** (0.75 lower to 0.23 lower) | ⨁⨁◯◯ LOW ^a,b,c^ | NSPT + Omega-3 PUFA (± ASA) may result in a reduction in Clinical Attachment loss at 3 months. |
| Clinical Attachment Loss at 6 months (follow-up: mean 6 months; assessed with: RevMan) | | | | | | | | | | | | |
| 7 | Randomized trials | serious^d^ | serious^e^ | not important | not important | Strongly suspected publication bias^c^ | 159 | 159 | - | MD **0.58 mm lower** (0.96 lower to 0.21 lower) | ⨁⨁◯◯ LOW ^c,d,e^ | NSPT + Omega-3 PUFA (± ASA) may result in a reduction in Clinical Attachment loss at 6 months. |
| Probing Pocket Depth at 3 months (follow-up: mean 3 months; assessed with: RevMan) | | | | | | | | | | | | |
| 10 | Randomized trials | serious^a^ | serious^f^ | not important | not important | -^g^ | 249 | 243 | - | MD **0.44 mm lower** (0.62 lower to 0.25 lower) | ⨁⨁⨁◯ MODERATE ^a,f,g^ | NSPT + Omega-3 PUFA (± ASA) may result in a reduction in Probing Pocket Depth at 3 months. |
| Probing Pocket Depth at 6 months (follow-up: mean 6 months; assessed with: RevMan) | | | | | | | | | | | | |
| 7 | Randomized trials | serious^d^ | serious^h^ | not important | not important | Strongly suspected publication bias^c^ | 159 | 159 | - | MD **0.45 mm lower** (0.76 lower to 0.14 lower) | ⨁⨁◯◯ LOW ^c,d,h^ | NSPT + Omega-3 PUFA (± ASA) may result in a reduction in Probing Pocket Depth at 3 months. |

**CI:** Confidence interval; **MD:** Mean difference

**Footnotes**

a. Six of the ten included studies were at low risk of bias, while four studies had unclear risk of bias. This introduces some uncertainty in the overall estimate, so the quality of evidence was downgraded by one level for study limitations (risk of bias).

b. The substantial heterogeneity (I² = 84%) may be explained by differences in intervention doses and patient populations across studies. For this reason, the quality of evidence was downgraded for inconsistency

c. The funnel plot indicates a possible presence of publication bias, as the distribution of studies is slightly skewed toward results favoring SRP + Omega-3. Consequently, the overall pooled effect might be somewhat overestimated, although the evidence is not conclusive due to high heterogeneity of included studies.

d. Four of the seven included studies were at low risk of bias, while three studies had unclear risk of bias. This introduces some uncertainty in the overall estimate, so the quality of evidence was downgraded by one level for study limitations (risk of bias)

e. The substantial heterogeneity (I² = 87%) may be explained by differences in intervention doses and patient populations across studies. For this reason, the quality of evidence was downgraded for inconsistency.

f. The substantial heterogeneity (I² = 81%) may be explained by differences in intervention doses and patient populations across studies. For this reason, the quality of evidence was downgraded for inconsistency.

g. Overall, no clear signs of publication bias emerge, although the limited number of studies reduces the tool’s ability to detect potential distortions.

h. The substantial heterogeneity (I² = 89%) may be explained by differences in intervention doses and patient populations across studies. For this reason, the quality of evidence was downgraded for inconsistency.

**Figure S2.** Funnel Plot – Clinical Attachment loss (CAL) at 3 months.


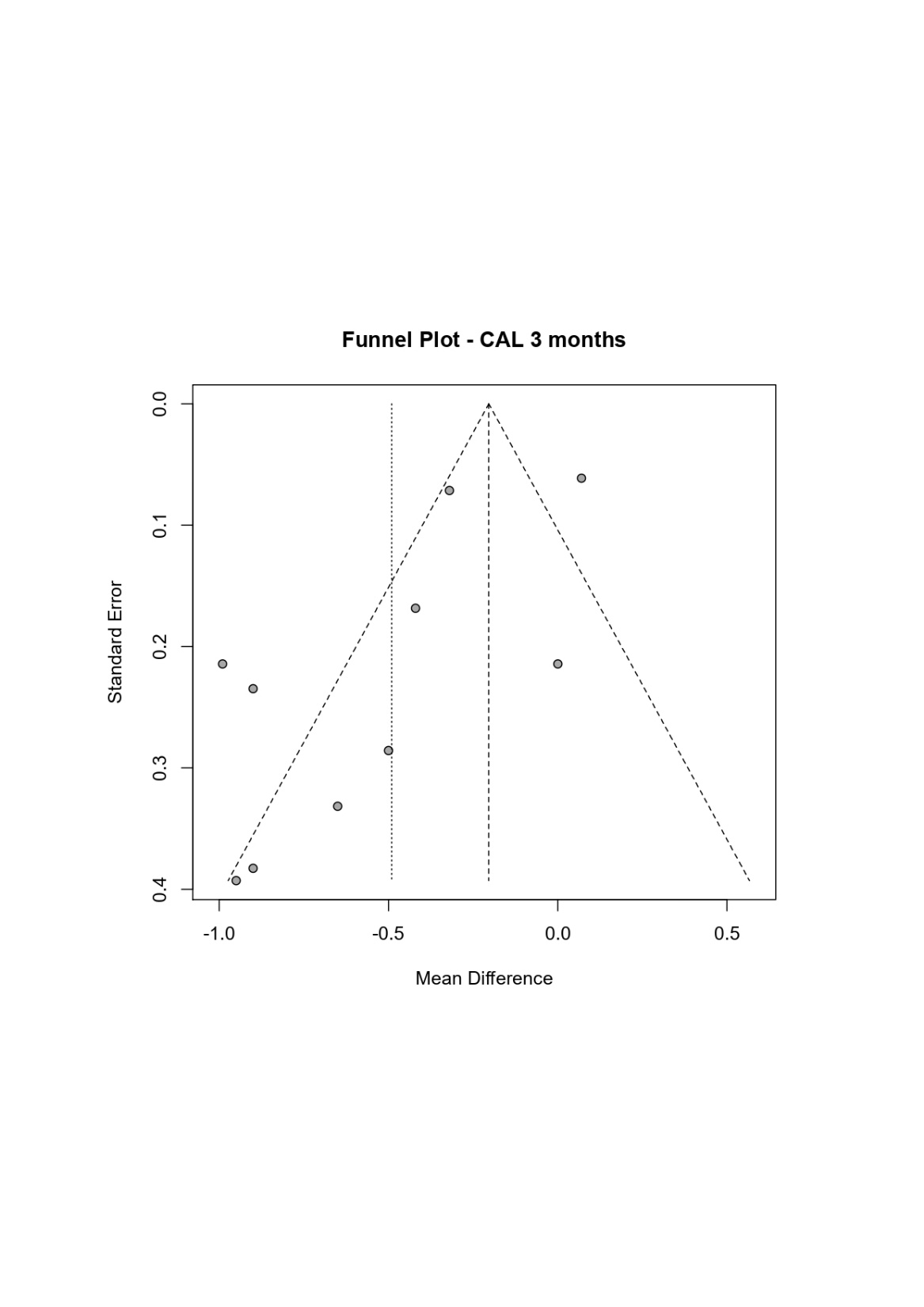


**Figure S3.** Funnel Plot – Clinical Attachment loss (CAL) at 6 months.


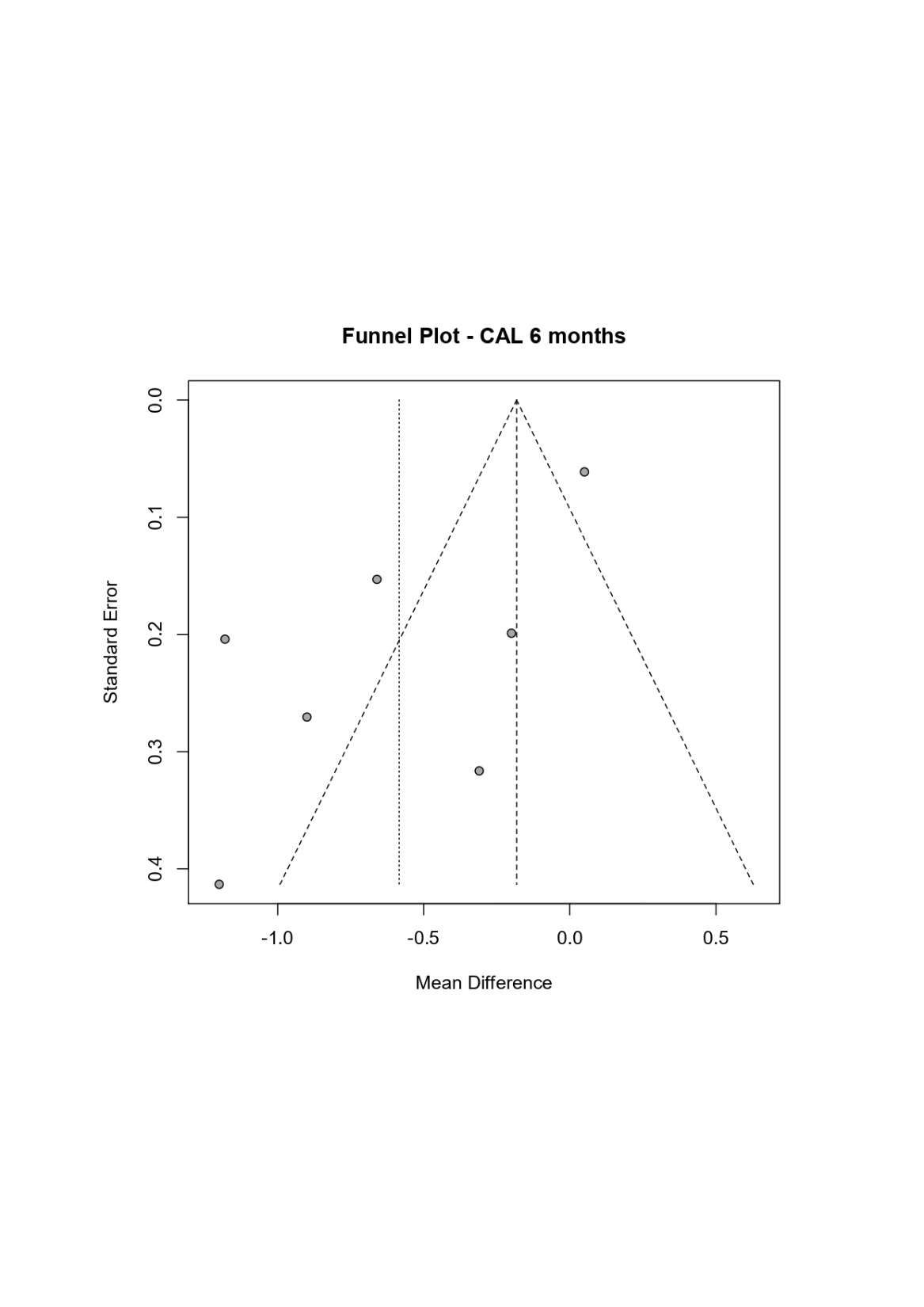


**Figure S4** Funnel Plot – Probing Pocket Depth (PPD) at 3 months.
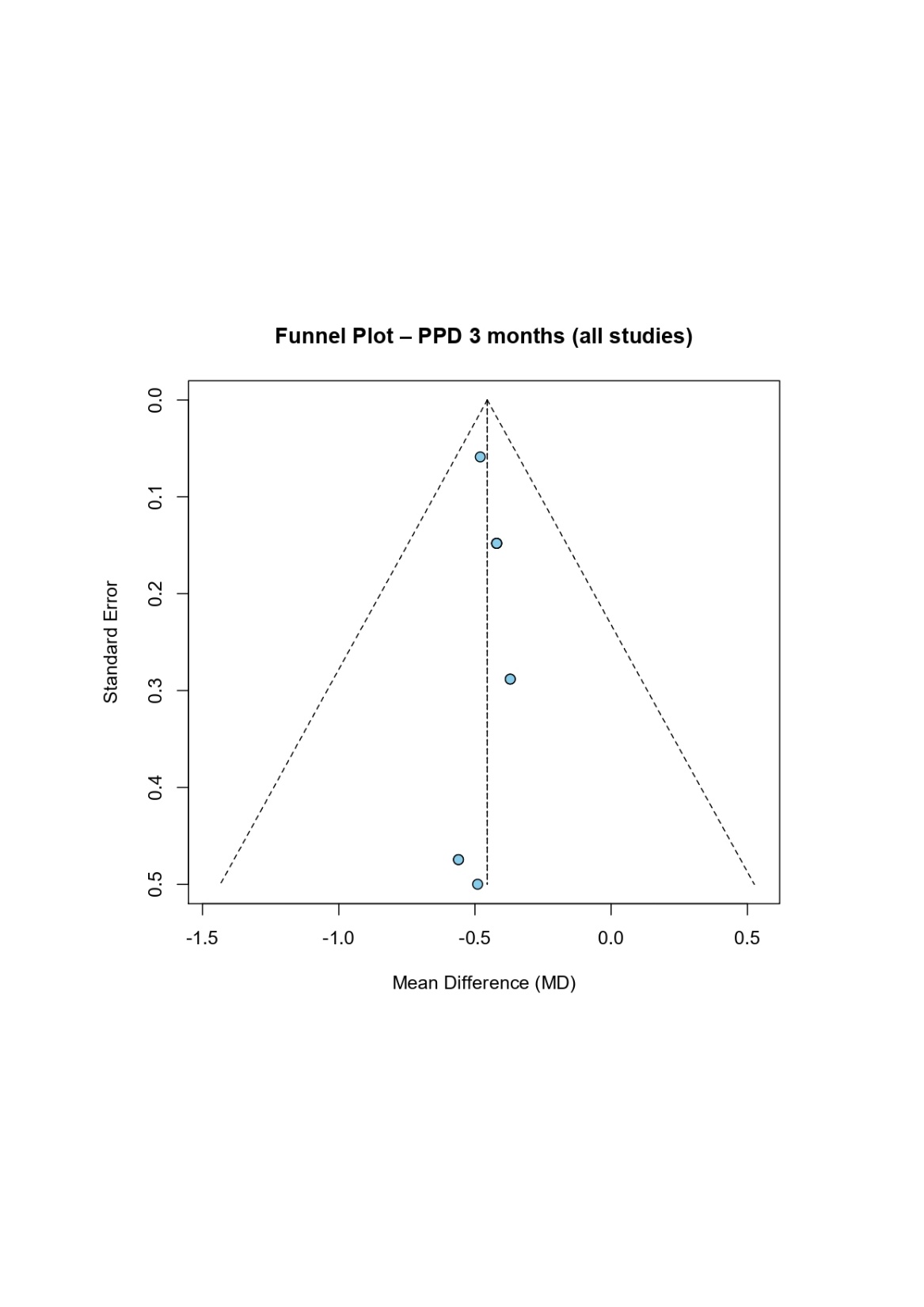


**Figure S5.** Funnel Plot – Probing Pocket Depth (PPD) at 6 months.


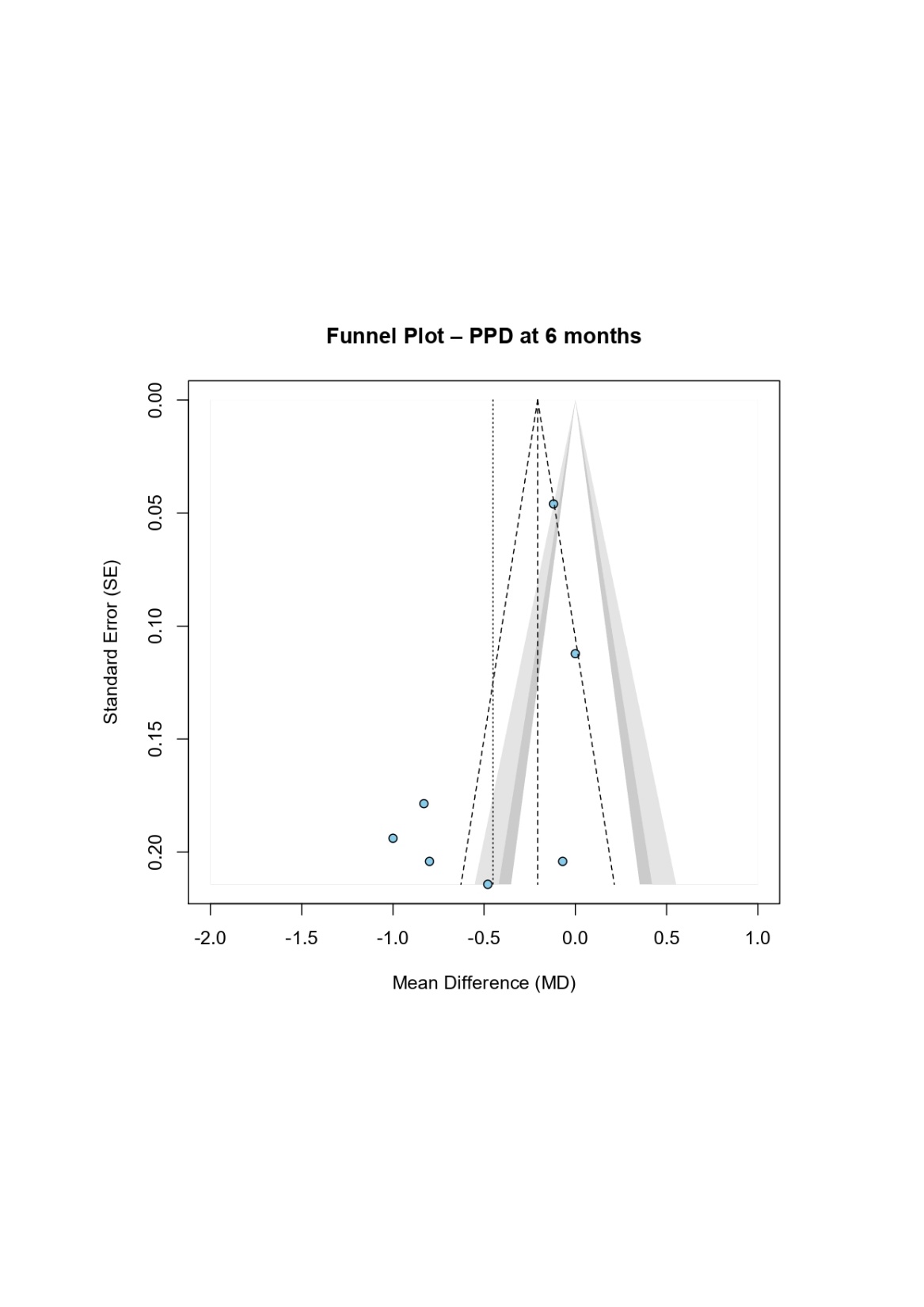

Supplement: Supplementary file 1 [file Datasheet1.docx]
